# Comparative Effects of Target-Controlled Infusion of Propofol Versus Spinal and Thiopental-Sevoflurane Anesthesia on Lipid Peroxidation in Elective Cesarean Section: A Prospective, Open-Label Study

**DOI:** 10.7759/cureus.61995

**Published:** 2024-06-09

**Authors:** Suzana Sobot Novakovic, Snezana Uletilovic, Nebojsa Mandic-Kovacevic, Tanja Cvjetkovic, Milos P Stojiljkovic, Ranko Skrbic, Dragana Loncar-Stojiljkovic

**Affiliations:** 1 Anesthesiology and Critical Care, University Clinical Centre of the Republic of Srpska, Banja Luka, BIH; 2 Anesthesiology and Critical Care, Centre for Biomedical Research, Faculty of Medicine, University of Banja Luka, Banja Luka, BIH; 3 Medical Biochemistry and Chemistry, Centre for Biomedical Research, Faculty of Medicine, University of Banja Luka, Banja Luka, BIH; 4 Pharmacy, Centre for Biomedical Research, Faculty of Medicine, University of Banja Luka, Banja Luka, BIH; 5 Pharmacology, Toxicology and Clinical Pharmacology, Centre for Biomedical Research, Faculty of Medicine, University of Banja Luka, Banja Luka, BIH; 6 Anesthesiology and Critical Care, Institute for Cardiovascular Diseases “Dedinje”, Belgrade, SRB; 7 Anesthesiology and Critical Care, Centre for Biomedical Research, Faculty of Medicine, University of Banja Luka, The Republic of Srpska, Banja Luka, BIH

**Keywords:** antioxidants, propofol, lipid peroxidation, oxidative stress, cesarean section, obstetric anesthesia

## Abstract

Background: During pregnancy, physiological changes can increase oxidative stress (OS) in both mothers and fetuses. The use of anesthesia for cesarean sections (CSs) could exacerbate this stress due to its impact on the ischemia-reperfusion effect. Our study aimed to explore the effects of target-controlled infusion of propofol on OS during CSs, and to compare these effects with those of spinal and thiopental-sevoflurane anesthesia.

Methods: The study included ninety parturients undergoing elective CS, allocated into three groups: Group S (spinal) (n = 30), Group P (propofol) (n = 30), and Group TS (thiopental-sevoflurane) (n = 30). Venous blood samples were taken from mothers at three time points, before, during, and after surgery, and one sample was taken from the umbilical vein after delivery. Blood samples were analyzed with the thiobarbituric acid reactive substances (TBARS) assay and blood gas analysis. A statistical comparison between groups was obtained by one-way analysis of variance (ANOVA) and the Wilcoxon test where appropriate.

Results: Levels of TBARS after the induction of anesthesia were lower in all groups compared to values preoperatively. In Group P, TBARS levels started to decrease in the first five minutes after the induction (1.90 ± 0.47; P < 0.001) and had significantly lower values compared to Group S (2.22 ± 0.21) and Group TS (2.40 ± 0.20). Two hours after surgery, TBARS values were the lowest in Group P (1.76 ± 0.15, P<0.001), compared to Group S (2.18 ± 0.24) and Group TS (2.41 ± 0.21). TBARS value in umbilical venous blood was significantly lower in Group P (1.56 ± 0.16, P < 0.001) compared to Group S (2.18 ± 0.17) and Group TS (2.09 ± 0.09). Umbilical cord venous blood gas values (pH, PCO_2_, HCO_3_, lactates, and base excess (BE)) were not different between the groups, except for PO_2_, which was significantly lower in Group S (20.5 ± 5.0; P < 0.001) compared to Group P (36.5 ± 19.2) and Group TS (33.5 ± 10.1).

Conclusion: Target-controlled infusion of propofol anesthesia could be advantageous for parturients with compromised oxidative status, especially those undergoing emergency CSs when general anesthesia is required.

## Introduction

Pregnancy is a condition that puts the expectant mother in a state of elevated oxidative stress (OS), with the placenta acting as a producer of oxygen free radicals [1]. Increased metabolic needs that allow adequate fetal growth and development are accompanied by enhanced OS in the placenta [2]. Certain factors make a fetus and a newborn even more vulnerable to OS compared to their mothers. During childbirth, a neonate moves from an oxygen-poor environment inside the uterus to an oxygen-rich environment outside. This shift is believed to increase the production of reactive oxygen species and reactive nitrogen species [3]. The lower concentration of antioxidants in fetal plasma could account for the increased OS in the newborn compared to the mother. It has been observed that vaginal delivery and emergency cesarean section (CS) are linked with higher levels of OS compared to elective CS [4,5]. One of the main goals of CS anesthesia is to ensure effective anesthesia during surgery while minimizing the harmful effects of the anesthetics on the mother and the baby. Therefore, understanding the impact of anesthesia on OS during CS could be crucial in minimizing additional stress on the mother and fetus during delivery.

Malondialdehyde (MDA) is a lipid peroxidation product and it has been used as an indirect measure of oxidative damage [6]. The thiobarbituric acid reactive substances (TBARS) assay can detect its levels in the systemic circulation. TBARS represents a marker of ischemia-reperfusion damage [7]. The antioxidant properties of propofol have been confirmed through many studies, including in vitro experiments, animal research, and human trials [8-11]. Several studies examined the effects of propofol on OS during general anesthesia for CS [12,13]. However, no studies investigated the impact of target-controlled infusion (TCI) of propofol during general anesthesia for CS on OS.

The main objective of this research is to investigate the impact of general and spinal anesthesia on OS during CS. We hypothesized that propofol anesthesia with TCI results in decreased OS for the mother and the fetus during CS compared to thiopental-sevoflurane anesthesia.

## Materials and methods

Study design and participants

This study was a prospective, open-label, comparative study. The study was approved by the Ethics Committee of the University Clinical Centre of the Republic of Srpska (UCC RS) (certificate No. 01-19-161-2\22, dated April 26, 2022) and the Ethics Committee of the Faculty of Medicine, University of Banja Luka (certificate No. 18\4. 244\22, dated September 22, 2022). The study was performed at the Clinic for Gynecology and Obstetrics, UCC RS, from November 2022 until February 2024. The blood samples were analyzed at the Centre for Biomedical Research, Faculty of Medicine, University of Banja Luka.

Following the obtaining of the written informed consent, the study included patients who had uncomplicated singleton pregnancies, with an estimated fetal weight exceeding 2,500 grams, undergoing an elective CS. Patients who underwent emergency CS, those with thrombophilia, pregnancy-induced hypertension (PIH), diabetes mellitus, smokers, cases of intrauterine fetal growth retardation (IUFGR), and fetal malformations were not included in the study.

Patients scheduled for elective CS were admitted to the hospital 24 hours prior to their surgery. On the day of admission, those who met the study’s inclusion criteria were approached. After reading the study information, informed consent was obtained from these patients. According to the hospital protocol, patients were provided with detailed information about the anesthesia options for their CS. They were then given the option to select their preferred method of anesthesia, spinal or general anesthesia. Those patients who opted for general anesthesia were randomly assigned to receive either target-controlled infusion (TCI) for propofol or thiopental-sevoflurane anesthesia. The randomization was conducted utilizing a simple method of randomization, specifically through the use of an online random number generator (https://www.calculatorsoup.com/), ensuring an unbiased and systematic allocation of subjects. After allocation study groups were formed: Group S, where patients received spinal anesthesia for CS; Group P, where patients received TCI of propofol for CS; and Group TS, where patients were administered thiopental for induction and sevoflurane for the maintenance of anesthesia.

Anesthesia procedures

All patients preoperatively received 500 ml of Hartman solution intravenously over one-two hours and were premedicated with metoclopramide 10 mg IV. In the operating room (OR), patients were placed on the operating table in the left lateral position. Two IV cannulas and non-invasive monitoring (blood pressure, ECG, SpO_2_) were placed. This was an open-label study so participants and researchers were aware of the type of anesthesia (general or spinal) that would be administered. Additionally, due to the inherent modalities of administration and the physicochemical properties of propofol and thiopental-sevoflurane, it was not possible to implement a blinded protocol.

In Group S, the spinal anesthesia was performed with 27G pencil point needles (Braun, Pencan). 2.2 ml of 0.5% levobupivacaine and 15 μg of fentanyl were injected intrathecally at L3-L4 or L4-L5 interspinous space. In Group P, the TCI pump (FRESENIUS KABI Agilia TIVA) was used for anesthesia induction and maintenance. The Schnider pharmacokinetic model was the preferred choice for patients. However, when its application was not feasible, the Marsh model was an effective alternative. In this group, the depth of anesthesia was controlled with bispectral index (BIS) monitoring. BIS electrodes were placed on the left side of the forehead (Covidien, Bispectral Index™ (BIS™) Monitoring System). Anesthesia was induced with propofol 4-6 μg/ml, doses adjusted according to BIS values, and succinylcholine 1.5 mg/kg for intubation. Patients were intubated when BIS values were targeted below 60 and ventilation was started with a 50% O_2_/air mixture. Atracurium was used for intraoperative relaxation as needed (0.5 mg/kg initial dose, 0.1 mg/kg for maintenance). Propofol doses for anesthesia maintenance were adjusted according to BIS (target values from 40 to 60). The alarms on the BIS device were set to alarm when the values fell below 40 and exceeded 60. In Group TS, anesthesia was induced with thiopental 5 mg/kg and succinylcholine 1.5 mg/kg. Patients were intubated and ventilation was continued with sevoflurane in 50% O_2_/N_2_O mixture. Atracurium was used for intraoperative relaxation as needed (0.5 mg/kg initial dose, 0.1 mg/kg for maintenance). End-tidal sevoflurane concentrations (EtSev) were monitored. Target concentrations were between 0.7 and 1.3 minimal alveolar concentration (MAC). The decrease in systolic blood pressure > 20% from baseline in all groups was considered hypotension and treated with IV phenylephrine in a dosage of 100 μg. All monitored parameters were recorded every two minutes in a manually written anesthesia chart.

Blood sampling procedures

Venous blood samples were taken from mothers at different time points, and one sample was taken from the umbilical vein after delivery. To measure MDA, samples were collected in tubes containing 3.2% Na-citrate. Samples were collected in 2 ml heparinized syringes for blood gas analysis (BGA). Samples were collected from mothers at various time points during the perioperative procedure: in the OR before induction to anesthesia, after delivery and placental removal and two hours after surgery. An umbilical blood sample was obtained after delivery, just before placental removal. The procedure involved clamping the umbilical cord and drawing blood from the umbilical vein. One sample of umbilical venous blood was taken for BGA, and the second was taken for measuring MDA.

Biochemical parameters determination

Plasma was separated by centrifugation for 10 minutes at 3,000 rpm. All samples were stored at -80°C until measurement. The index of lipid peroxidation, TBARS, was determined by using 1% thiobarbituric acid (TBA) and 0.05M NaOH and measured at 530 nm.

Statistical analysis

Statistical analysis was performed with IBM Corp. Released 2021. IBM SPSS Statistics for Windows, Version 28.0 (Armonk, NY, IBM Corp). A statistical comparison between groups was obtained by one-way analysis of variance (ANOVA) with Bonferroni correction. Post-hoc analysis for ANOVA was performed using the Wilcoxon test. Descriptive statistical values were expressed as mean ± SD. P < 0.05 was considered statistically significant.

## Results

A total of ninety-one patients were initially enrolled in the study. One patient was excluded because of a failed spinal anesthesia. The study included ninety patients, with 30 patients in each group. The demographic data and APGAR scores are provided in Table 1. No significant differences were observed among the groups within the study population

**Table 1:** Demographic maternal characteristics, neonatal characteristics, and APGAR scores p-value significance at <0.05. Data are expressed as mean ± SD. n: Number of patients per group; AS 1: APGAR score in first minute; AS 5: Apgar score in fifth minute; ns: Not significant (P > 0.05); S: Spinal group; P: Propofol group; TS: Thiopental-sevoflurane group

	S (n = 30)	P (n = 30)	TS (n = 30)	P
Mothers				
Age	32.5 ± 4.9	31.9 ± 4.2	31.1 ± 5.3	ns
Height (cm)	168.2 ± 7.3	168.1 ± 5.7	168.2 ± 5.6	ns
Weight (kg)	79.4 ± 11.1	82.2 ± 13.9	82.8 ± 13.5	ns
BMI (kg/m^2^)	28.1 ± 3.5	29.2 ± 4.5	29.1 ± 5.3	ns
Gestation	38.6 ± 0.85	38.7 ± 0.82	38.7 ± 0.73	ns
Neonates				
Weight (g)	3,379.3 ± 544.2	3,423.7 ± 441.1	3,435 ± 402.5	ns
Length (cm)	52.1 ± 2.6	52.5 ± 2.1	52.3 ± 2.1	ns
AS 1	9.2 ± 0.4	9.1 ± 0.6	9.0 ± 0.4	ns
AS 5	9.8 ± 0.4	9.8 ± 0.4	9.7 ± 0.5	ns

The details of the surgical procedure are outlined in Table 2. The interval from induction to the start of the operation (IO), induction to delivery (ID), and the overall duration of anesthesia (DA) were notably extended in Group S (P < 0.001).

**Table 2:** Time points during surgery Values in cells represent mean time points expressed in minutes. All values are expressed as mean ± SD. n: Number of patients per group; IO: Induction to start of the operation; ID: Induction to delivery; OD: Start of the operation to delivery; DS: Duration of surgery; DA: Duration of anesthesia; S: Spinal group; P: Propofol group; TS: Thiopental-sevoflurane group ^***^ P <0 .001 versus P; ^†††^ P < 0.001 versus TS

Groups/Time Points	IO	ID	OD	DS	DA
S (n = 30)	5.3 ±1.9^***†††^	9.5 ± 2.7^***†††^	4.2 ± 1.7	41.4 ± 11.3	158.9 ± 26.5^***†††^
P (n = 30)	1.3 ± 0.8	4.6 ± 2.2	3.3 ± 1.9	48.1 ± 11.9	54.6 ± 12.9
TS (n = 30)	1.2 ± 0.4	4.3 ± 1.2	3.1 ± 1.6	45.6 ± 12.1	51.9 ± 11.4

During anesthesia mean arterial pressure (MAP) and heart rate (HR) were monitored at two-minute intervals until delivery. In Group S, the MAP was significantly lower at the second minute (84.5 ± 12.6, P < 0.001) and the fourth minute (79.8 ± 14.7, P < 0.001) of anesthesia compared to Group P (112.2 ± 17.7; 101.9 ± 16.1) and Group TS (105.4 ± 18.5; 96.3 ± 11.9) (Table 3). 

**Table 3:** MAP during surgery at different time points Values in cells represent MAP in mmHg. All values are expressed as mean ± SD. Numbers 0-10 represent the time in minutes from anesthesia induction; 0 represents values of MAP before anesthesia induction. n: Number of patients per group; S: Spinal group; P: Propofol group; TS: Thiopental-sevoflurane group; MAP: Mean arterial pressure ^***^ P <0 .001 versus P; ^††† ^P < 0.001 versus TS

Groups/Minutes	0	2	4	6	8	10
S (n=30)	91.5 ± 9.5	84.5±12.6^***†††^	79.8 ± 14.7^***†††^	82.7 ± 13.5	81.9 ± 12.7	81.7 ± 12.9
P (n=30)	95.1 ± 11.6	112.2 ± 17.7	101,.9 ± 16.1	89.6 ± 14.7	83.1 ± 11.4	82 ± 11.4
TS (n=30)	92.7 ± 11.9	105.4 ± 18.5	96.3 ± 11.9	85.4 ± 12.5	80.4 ± 12.2	80.4 ± 12.2

In a similar trend, the HR in Group S was also significantly lower at the second minute (82.1 ± 21.9, P <0.001) and the fourth minute (79.2 ± 17.4, P < 0.01) compared to Group P (103.7 ± 21.5; 95.6 ± 23.1) and Group TS (111.4 ± 18.7; 97.1 ± 20.5) (Table 4).

**Table 4:** HR during surgery Values in cells represent HR in beats per minute. All values are expressed as mean ± SD. Numbers 0-10 represent the time in minutes from anesthesia induction. 0 represents values of HR before anesthesia induction. n: Number of patients per group; S: Spinal group; P: Propofol group; TS: Thiopental-sevoflurane group; HR: Heart rate ^***^ P < 0 .001 versus P; ^††† ^P < 0.001 versus TS; ^**^ P <0.01 versus P; ^††^ P < 0.01 versus TS

Groups/Minutes	0	2	4	6	8	10
S (n=30)	87.8 ± 18.1	82.1±21.9^***†††^	79.2 ± 17.4^**††^	75.5 ± 16.1	82.6 ±15.1	78.2 ± 12.8
P (n=30)	86.5 ± 14.5	103.7 ±21.5	95.6 ±23.1	87.2 ± 15.1	82.5 ± 16.1	79.5 ± 11.4
TS (n=30)	93.2 ± 17.5	111.4 ± 18.,7	97.1 ± 20.5	84.7 ± 13.9	81.3 ± 13.9	80.1 ± 13.4

Umbilical cord BGA are presented in Table 5. Blood oxygenation values (pO_2_) were significantly lower in samples from Group S (20.5 ± 5.0, P <0.001) compared to Group P (36.5 ± 19.2) and Group TS (33.5 ± 10.1). The values of pH, pCO_2_, bicarbonates, and lactates were not different among groups (Table 5).

**Table 5:** Umbilical venous BGA All values are expressed as mean ± SD. BGA: Blood gas analysis; BE: Base excess; n: number of patients per group; S: Spinal group; P: Propofol group; TS: Thiopental-sevoflurane group ^***^ P < 0 .001 versus P; ^†††^ P < 0.001 versus TS

	S (n = 30)	P (n = 30)	TS (n = 30)
pH	7.34 ± 0.05	7.32 ± 0.03	7.32 ± 0.03
pCO_2_ (mmHg)	43.7 ± 5.4	45.4 ± 6.3	47.2 ± 4.9
pO_2_ (mmHg)	20.5 ± 5.0^***†††^	36.5 ± 19.2	33.5 ± 10.1
HCO_3_std (mmol/l)	21.4 ± 1.5	21.5 ± 1.2	21.9 ± 1.5
HCO_3_akt (mmol/l)	23.3 ± 1.8	23.3 ± 2.2	24.2 ± 2.3
BE (mmol/l)	-2.4 ± 1.9	-2.7 ± 1.7	-2.2 ± 1.9
Lactates (mmol/l)	1.8 ± 0.4	1.6 ± 0.4	1.6 ± 0.3

TBARS analysis

**Figure 1:**
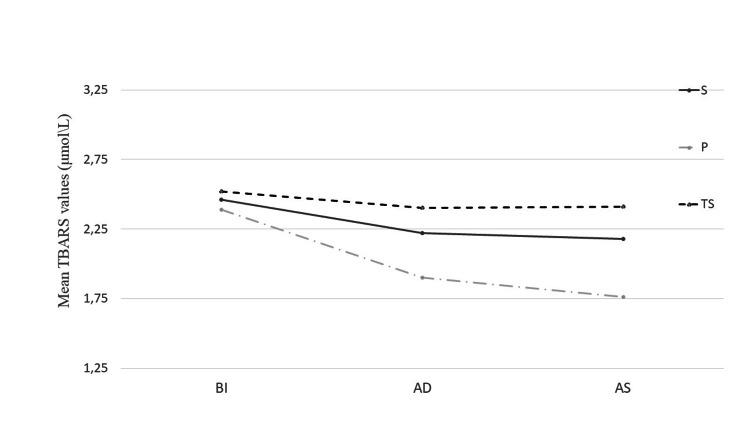
TBARS BI and after induction to anesthesia TBARS levels BI, AD to anesthesia, and AS among groups. TBARS levels AI to anesthesia decreased in all groups. A statistically significant decrease compared to BI levels was in Group S (P < 0.001) and Group P (P < 0.001), while in Group TS this decrease was not significant (P > 0.05). S: Spinal group; P: Propofol group; TS: thiopental-sevoflurane group; BI: Before induction; AD: After delivery; AS: After surgery

**Figure 2:**
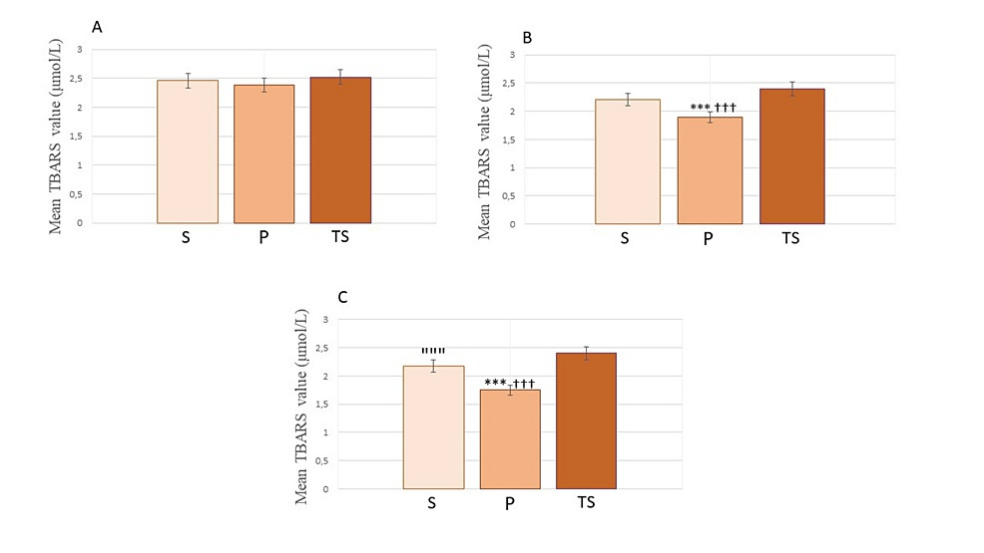
TBARS levels in mothers' blood at different time points before, during and after surgery A) Plasma values of TBARS before induction to anesthesia among groups. P > 0.05, no differences among groups; B) Plasma values of TBARS in parturients' blood during anesthesia and after delivery among groups; C) Plasma values of TBARS in mothers' blood after surgery among groups. All values are expressed as mean ± SD. S: Spinal group; P: Propofol group; TS: Thiopental-sevoflurane group ^***^ P< 0.001 compared to S; ^†††^ P< 0.001 compared to TS; ^”””^ P< 0.001 compared to TS

TBARS as a marker of lipid peroxidation was measured at several time points in the blood of parturients, and in one sample of umbilical venous blood. The mean values of TBARS before induction to anesthesia were without major differences among the groups (Group S - 2.46 ± 0.17; Group P - 2.40 ± 0.34; Group TS - 2.52 ± 0.18) (P > 0.05). TBARS levels measured after delivery showed that values have decreased in all groups compared to their preoperative values, but without significance in Group TS (P > 0.05) (Figure 1). The most significant decrease among groups after delivery was in the blood of the patients in Group P (1.90 ± 0.47; P< 0.001) compared to Group S (2.22 ± 0.21) and Group TS (2.40 ± 0.20). Two hours after surgery, TBARS values in blood of parturients continued to decrease in Group P (1.76 ± 0.15, P < 0.001), had a moderate decrease in Group S (2.18 ± 0.24) and stayed relatively unchanged in Group TS (2.41 ± 0.21). The decrease in TBARS levels was more significant in Group S than in Group TS (P < 0.001) (Figure 2).

**Figure 3:**
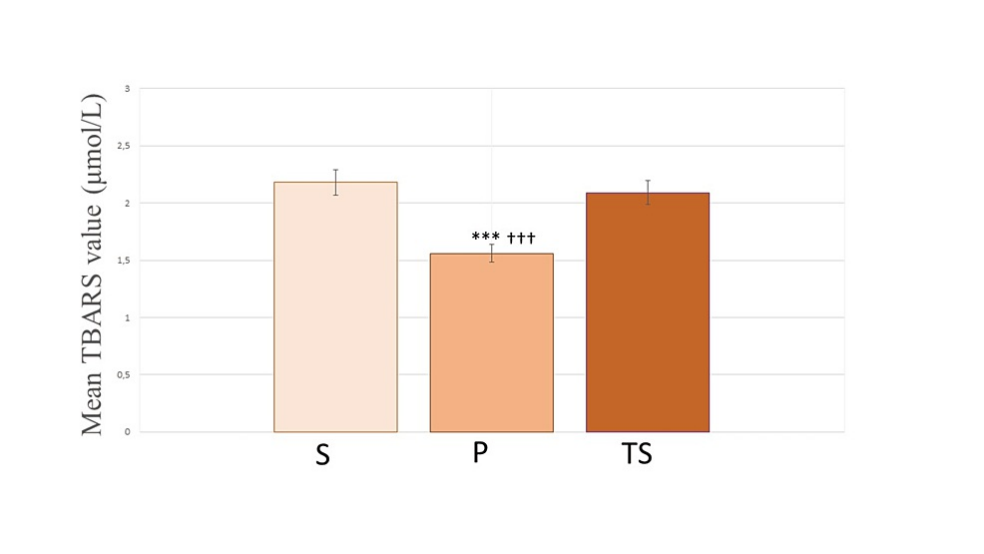
TBARS values in umbilical venous blood after delivery Plasma values of TBARS in umbilical cord venous blood among groups. All values are expressed as mean ± SD. S: Spinal group; P: Propofol group; TS: Thiopental-sevoflurane group ^***^ P< 0.001 compared to S; ^†††^ P< 0.001 compared to TS

The mean values of TBARS in umbilical venous blood were significantly lower in Group P (1.56 ± 0.16, P < 0.001) compared to Group S (2.18 ± 0.17) and Group TS (2.09 ± 0.09). No significant differences were found between Group S and Group TS (Figure 3).

## Discussion

In this study, we aimed to examine the effects of general and spinal anesthesia on OS in mothers and fetuses during elective CS. We measured lipid peroxidation by-products with the TBARS assay in blood samples from mothers and the umbilical cords of newborns. We hypothesized that anesthesia with TCI propofol reduces lipid peroxidation in both the mother and the placenta. This study demonstrates that TCI propofol anesthesia lowers the production of TBARS when compared to spinal and thiopental-sevoflurane anesthesia, confirming our hypothesis. Our data showed that mothers and neonates in Group P had the lowest levels of TBARS compared to Group S and Group TS. TBARS levels among mothers in Group P decreased in the first minutes of the anesthesia induction and continued to decrease even after surgery, whereas TBARS levels in the other two groups stayed relatively close to the values measured preoperatively. We confirmed that the effects of propofol on lipid peroxidation start very quickly, within the first five minutes from the start of the infusion, and their effect lasts even two hours after the surgery. Aldemir et al., in their study, investigated the effects of propofol on MDA production in knee arthroplasty [14]. Similar to our study, they concluded that the effects of propofol were decreasing MDA in the propofol group five minutes after the tourniquet was placed, and even one and five minutes after the release of the tourniquet. They explained these results with the theory that tissues beneath the tourniquet are believed to be infused with propofol. Therefore, propofol, either attached to proteins or located within membranes, could potentially inhibit the generation of free radicals and the ensuing lipid peroxidation that might occur after the tourniquet release.

In our study, in contrast to the group that received propofol, the spinal group, and the thiopental-sevoflurane group had a mild decrease in TBARS after induction. Two hours after surgery, their levels stayed very close to the ones after anesthesia induction. Thiopental and sevoflurane, in previous studies, showed some antioxidant effects but were much lesser compared to propofol [15,16]. This explains their very mild antioxidant effects in the current study. However, in spinal anesthesia contrasting data about its effect on OS in mothers and fetuses were reported. Okudaira et al. hypothesized that ischemia-reperfusion in fetuses leads to increased production of oxypurines (xanthine and uric acid) and lipid peroxides (MDA), which proved to be increased in pregnant patients under spinal anesthesia who had hypotension lasting longer than two minutes [17]. Karabayırlı et al. compared total antioxidant status, total oxidant status, and oxidative stress index (OSI) levels in the umbilical arterial blood of pregnant women undergoing CS under general anesthesia with propofol, spinal, and epidural anesthesia. The OSI was significantly lower in the group with general anesthesia using propofol compared to the spinal and epidural groups [12]. Akin et al. investigated how general and regional anesthesia affects the level of thiol-disulfide (TDH), a marker of OS, in mothers and their newborns. General anesthesia was performed with propofol and sevoflurane, while spinal anesthesia was performed with standard doses of hyperbaric bupivacaine. Their findings indicated that the use of general anesthesia in CS decreases TDH when contrasted with spinal anesthesia, meaning more OS in groups with general anesthesia [13]. In our study, spinal anesthesia mildly decreased TBARS after induction, and those values stayed relatively the same two hours after the surgery. In conclusion, all three types of anesthesia lowered TBARS production, but that effect was the most prominent in the propofol group.

Studies have already confirmed that propofol has an antioxidant effect [18,19]. However, its use as a continuous infusion during CS is not well established. Few studies examine the use of continuous infusion of propofol and its safety profile during CS. Van de Velde et al. conducted a study examining the maternal and neonatal effects of target-controlled infusion of propofol and remifentanil for CSs. The study was carried out in a small number of patients (n=10), tracking the hemodynamic effects on the mother intraoperatively, and the Apgar scores of the newborns. The study concluded that the technique is safe, although recommendations for further research were made [20]. Similarly, Hu et al. conducted a study examining the effects of total intravenous anesthesia (TIVA) with propofol and remifentanil on the mother and newborn, and compared the safety in groups that had different times from ID. The study found no significant differences among the groups, confirming the safety of the technique [21].

Previous studies have noted that anesthesia for CS could potentially increase OS due to induced hypotension or desaturation [12,13,17]. According to Calderon et al., production of plasma purines and MDA was higher in vaginal delivery augmented with oxytocin compared to elective CS [22]. Additionally, researchers observed higher MDA levels in neonates born via emergency CS compared to those born during elective CS [23]. Given the previously discussed factors, it is crucial to understand the formation of free radicals during CS, the impact of anesthesia on their production, and the resulting effects on both the mother and the fetus. This understanding will help devise strategies to lower OS levels and mitigate the detrimental effects of this process on both the mother and the newborn.

This study has potential limitations. One significant limitation of this study is the absence of randomization and the open-label character of the study where anesthesia treatment was not blinded for participants nor researchers. The participants were not randomly assigned to the different groups, which could potentially introduce bias and affect the generalizability of the results. This lack of randomization may have led to an imbalance in the distribution of certain confounding variables across the groups. The study did not employ randomization due to ethical considerations. According to hospital protocols, pregnant patients scheduled for elective CS have the right to select their preferred type of anesthesia, provided there are no contraindications. Only those patients who opted for general anesthesia were randomly assigned to either the propofol group or the thiopental-sevoflurane group. This is a study limitation that should be considered when interpreting the results. Also, one other potential study’s limitation is that it is focused on patients undergoing elective CSs. This controlled condition may introduce selection bias, as it does not account for patients who experienced emergency CSs making impossible for comparation and to create further conclusions. Future research should consider including a more diverse patient population to enhance the generalizability of findings. Hypotension during spinal anesthesia could possibly be confounding factor. The study has a small sample size, which may lead to less reliable results.

## Conclusions

In summary, our study has confirmed the antioxidative effects of propofol in pregnant patients during elective CS. These effects could be particularly beneficial in emergency CS characterized by increased OS. However, it is essential to emphasize that while propofol demonstrates these benefits, general anesthesia with propofol should not be used as a first-choice anesthesia for CS. Spinal anesthesia remains the gold standard and should be the primary choice for CS. Nevertheless, in cases where general anesthesia is indicated, TCI with propofol should be preferred over thiopental-sevoflurane anesthesia due to its potential advantages, especially for emergency CS where the oxidative status of mother and fetus is compromised. Further research is required to confirm these findings and explore the potential benefits of propofol in this context.
